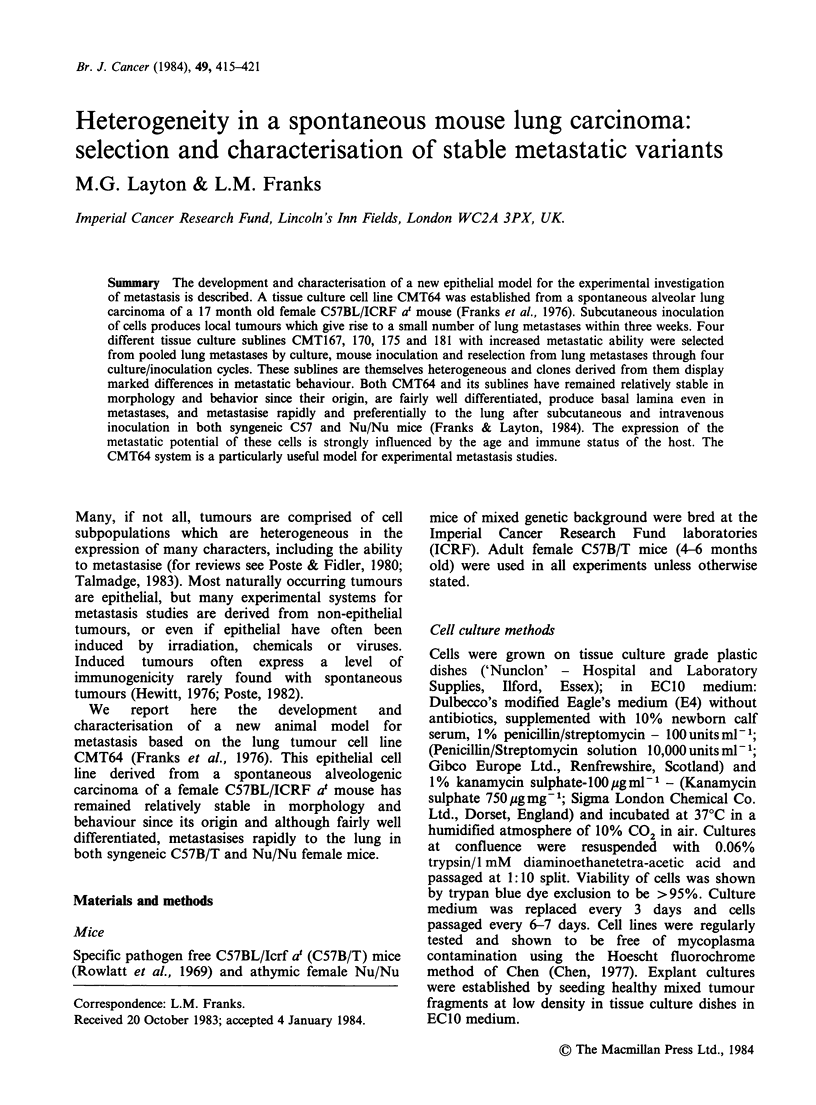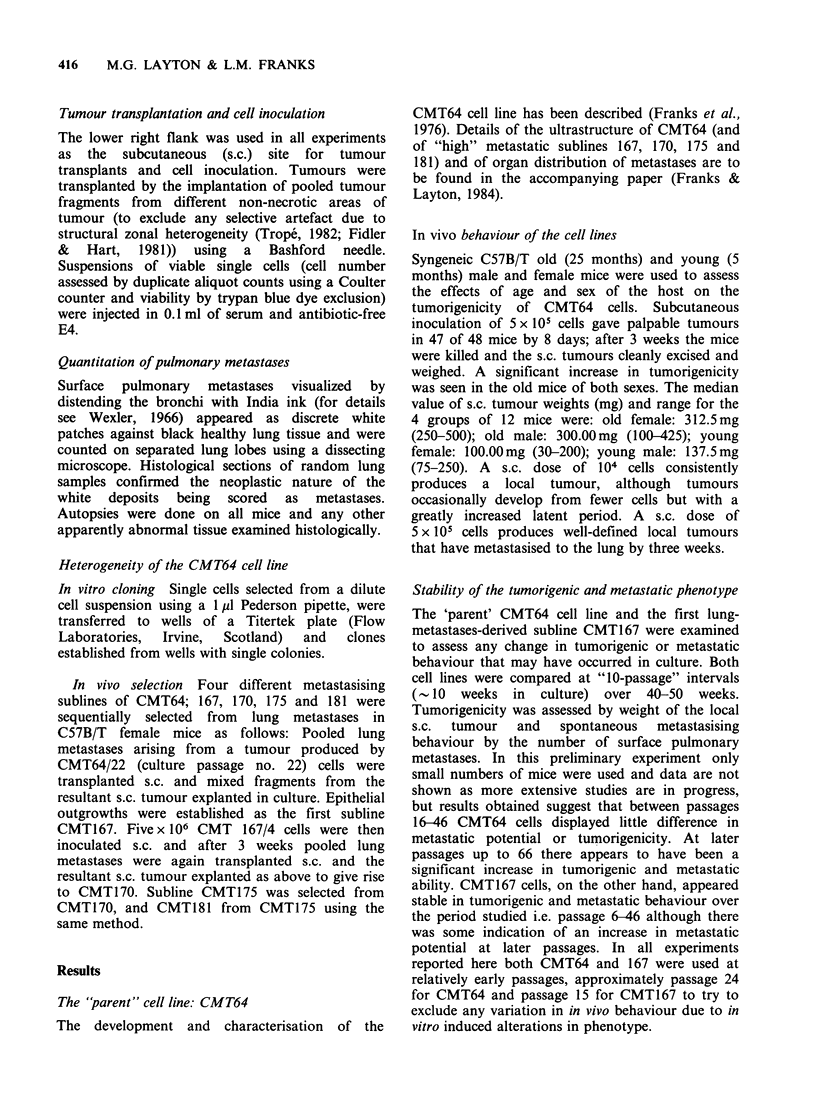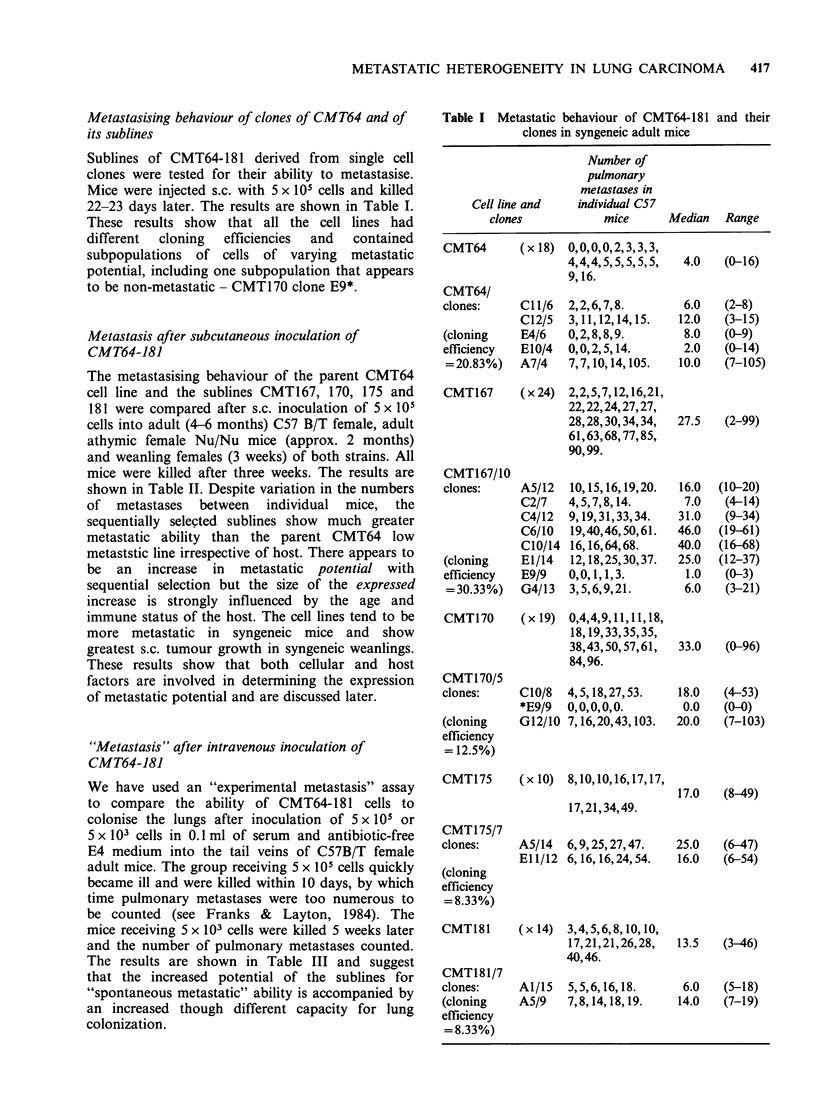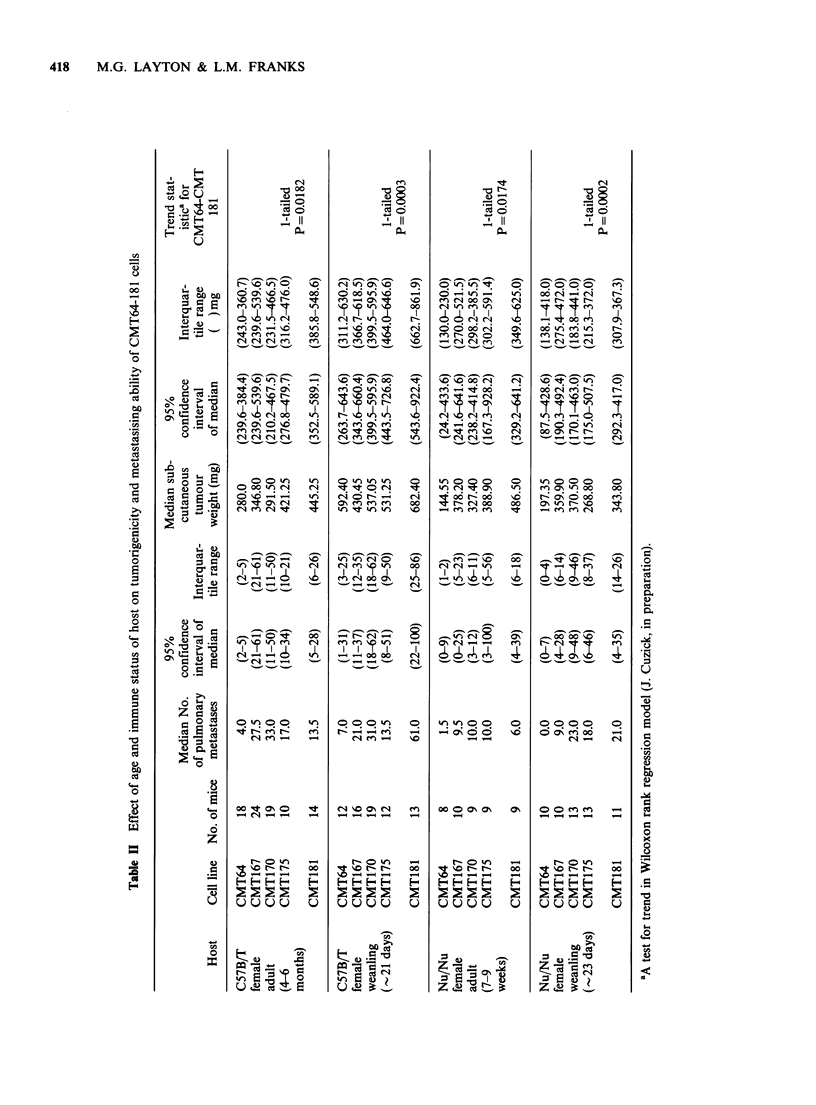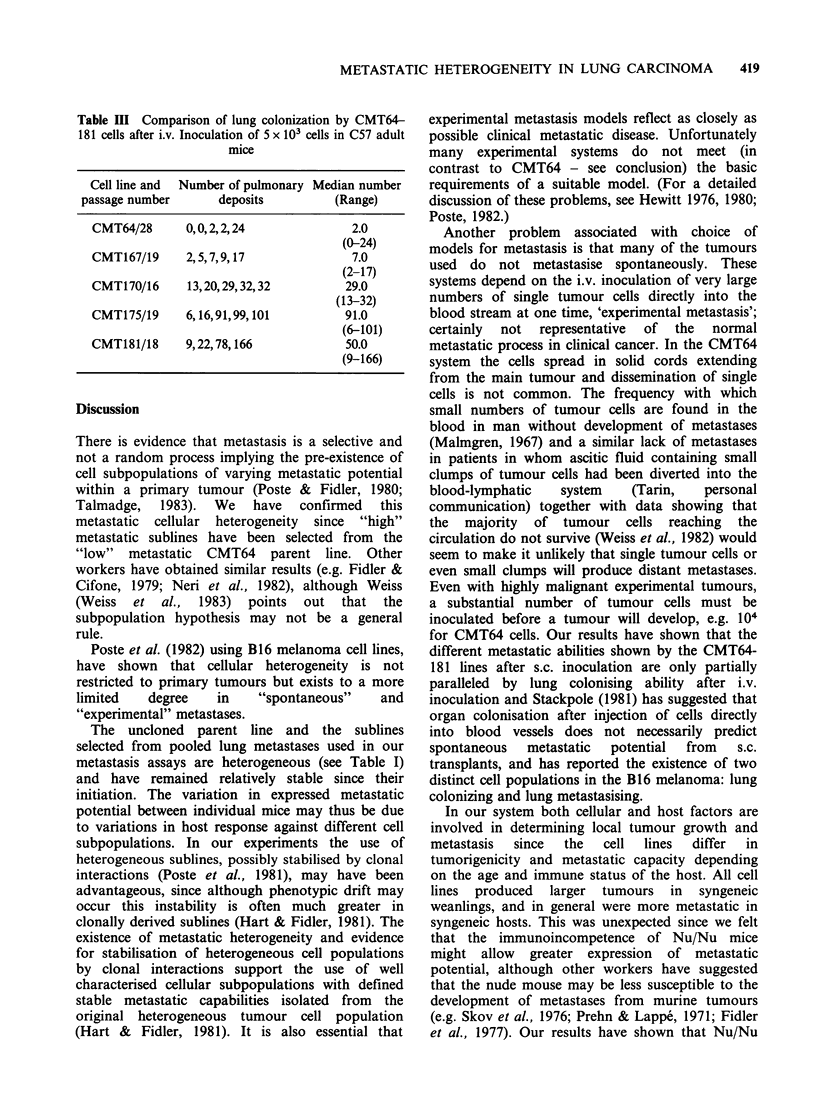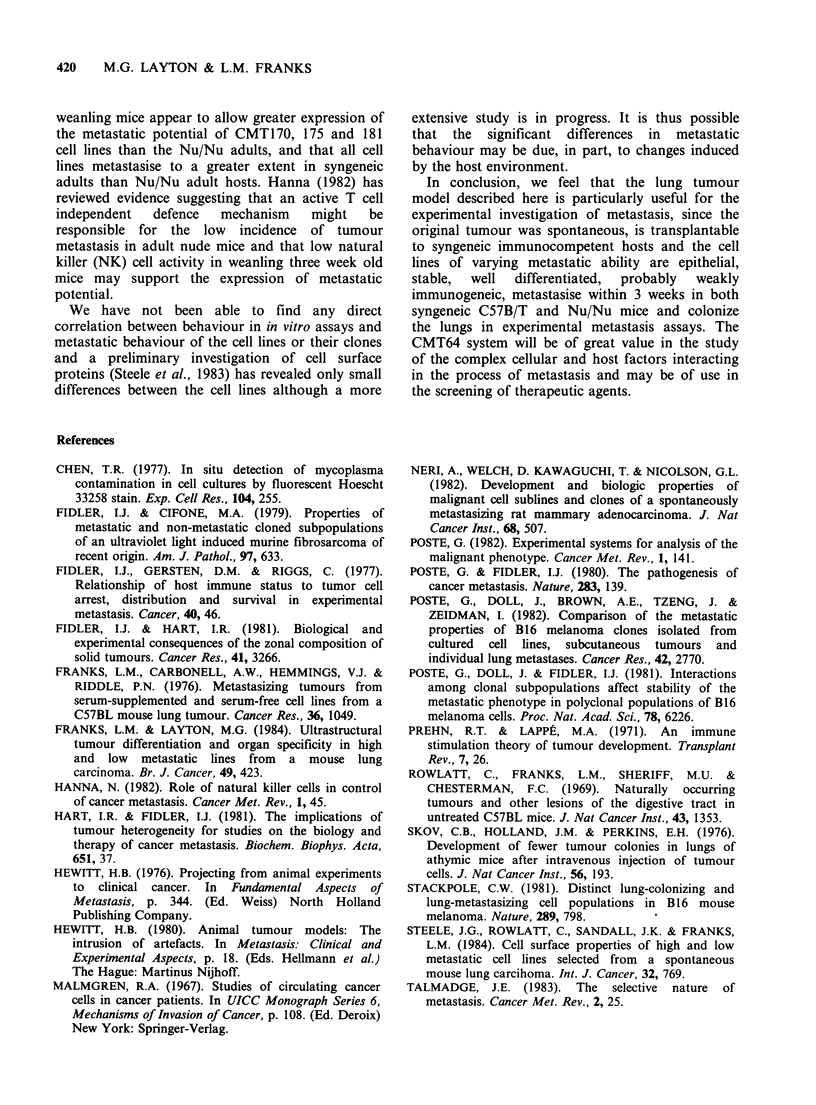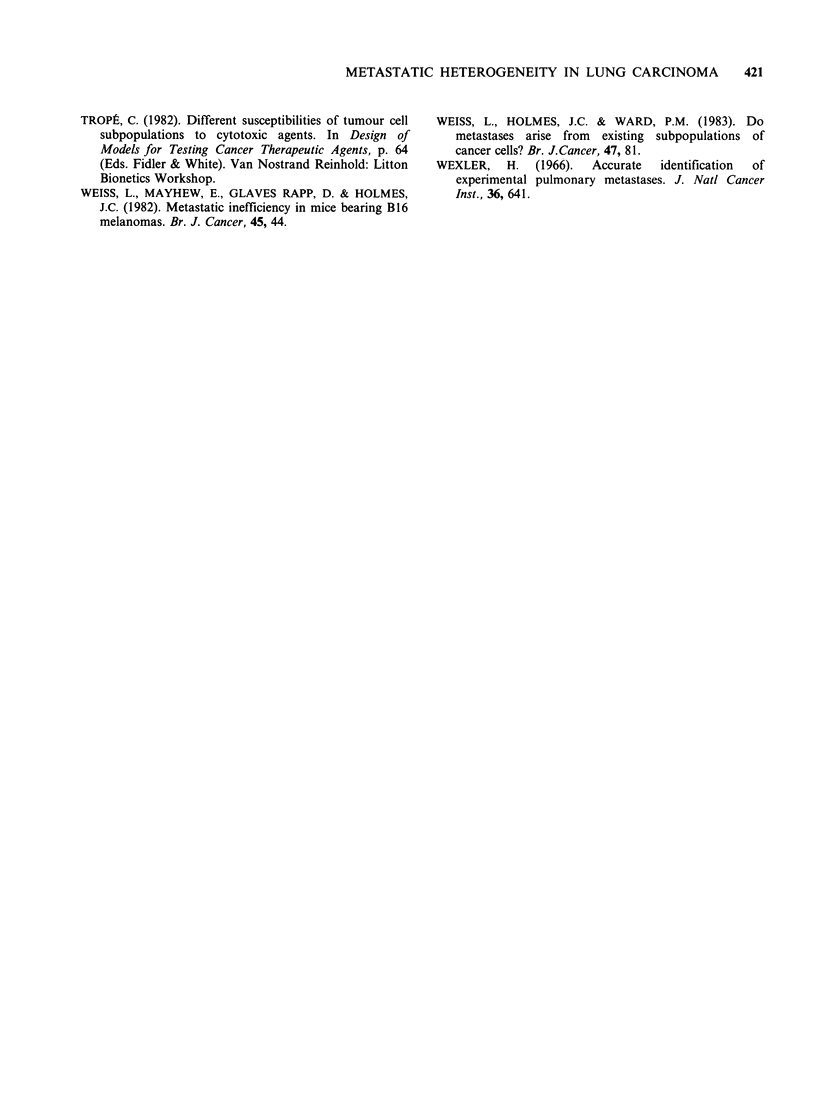# Heterogeneity in a spontaneous mouse lung carcinoma: selection and characterisation of stable metastatic variants.

**DOI:** 10.1038/bjc.1984.67

**Published:** 1984-04

**Authors:** M. G. Layton, L. M. Franks

## Abstract

The development and characterisation of a new epithelial model for the experimental investigation of metastasis is described. A tissue culture cell line CMT64 was established from a spontaneous alveolar lung carcinoma of a 17 month old female C57BL/ICRF at mouse (Franks et al., 1976). Subcutaneous inoculation of cells produces local tumours which give rise to a small number of lung metastases within three weeks. Four different tissue culture sublines CMT167, 170, 175 and 181 with increased metastatic ability were selected from pooled lung metastases by culture, mouse inoculation and reselection from lung metastases through four culture/inoculation cycles. These sublines are themselves heterogeneous and clones derived from them display marked differences in metastatic behaviour. Both CMT64 and its sublines have remained relatively stable in morphology and behavior since their origin, are fairly well differentiated, produce basal lamina even in metastases, and metastasise rapidly and preferentially to the lung after subcutaneous and intravenous inoculation in both syngeneic C57 and Nu/Nu mice (Franks & Layton, 1984). The expression of the metastatic potential of these cells is strongly influenced by the age and immune status of the host. The CMT64 system is a particularly useful model for experimental metastasis studies.


Br. J. Cancer (1984), 49, 415–421

### Heterogeneity in a spontaneous mouse lung carcinoma: selection and characterisation of stable metastatic variants

M.G. Layton & L.M. Franks

*Imperial Cancer Research Fund, Lincoln's Inn Fields, London WC2A 3PX, UK.*

**Summary** The development and characterisation of a new epithelial model for the experimental investigation of metastasis is described. A tissue culture cell line CMT64 was established from a spontaneous alveolar lung carcinoma of a 17 month old female C57BL/ICRF $a^t$ mouse (Franks *et al.*, 1976). Subcutaneous inoculation of cells produces local tumours which give rise to a small number of lung metastases within three weeks. Four different tissue culture sublines CMT167, 170, 175 and 181 with increased metastatic ability were selected from pooled lung metastases by culture, mouse inoculation and reselection from lung metastases through four culture/inoculation cycles. These sublines are themselves heterogeneous and clones derived from them display marked differences in metastatic behaviour. Both CMT64 and its sublines have remained relatively stable in morphology and behavior since their origin, are fairly well differentiated, produce basal lamina even in metastases, and metastasise rapidly and preferentially to the lung after subcutaneous and intravenous inoculation in both syngeneic C57 and Nu/Nu mice (Franks & Layton, 1984). The expression of the metastatic potential of these cells is strongly influenced by the age and immune status of the host. The CMT64 system is a particularly useful model for experimental metastasis studies.

Many, if not all, tumours are comprised of cell subpopulations which are heterogeneous in the expression of many characters, including the ability to metastasise (for reviews see Poste & Fidler, 1980; Talmadge, 1983). Most naturally occurring tumours are epithelial, but many experimental systems for metastasis studies are derived from non-epithelial tumours, or even if epithelial have often been induced by irradiation, chemicals or viruses. Induced tumours often express a level of immunogenicity rarely found with spontaneous tumours (Hewitt, 1976; Poste, 1982).

We report here the development and characterisation of a new animal model for metastasis based on the lung tumour cell line CMT64 (Franks *et al.*, 1976). This epithelial cell line derived from a spontaneous alveologenic carcinoma of a female C57BL/ICRF $a^t$ mouse has remained relatively stable in morphology and behaviour since its origin and although fairly well differentiated, metastasises rapidly to the lung in both syngeneic C57B/T and Nu/Nu female mice.

### Materials and methods

### *Mice*

Specific pathogen free C57BL/Icrf $a^t$ (C57B/T) mice (Rowlatt *et al.*, 1969) and athymic female Nu/Nu mice of mixed genetic background were bred at the Imperial Cancer Research Fund laboratories (ICRF). Adult female C57B/T mice (4–6 months old) were used in all experiments unless otherwise stated.

### *Cell culture methods*

Cells were grown on tissue culture grade plastic dishes ('Nunclon' – Hospital and Laboratory Supplies, Ilford, Essex); in EC10 medium: Dulbecco's modified Eagle's medium (E4) without antibiotics, supplemented with 10% newborn calf serum, 1% penicillin/streptomycin – 100 units $ml^{-1}$; (Penicillin/Streptomycin solution 10,000 units $ml^{-1}$; Gibco Europe Ltd., Renfrewshire, Scotland) and 1% kanamycin sulphate-100 $\mu g\,ml^{-1}$ – (Kanamycin sulphate 750 $\mu g\,mg^{-1}$; Sigma London Chemical Co. Ltd., Dorset, England) and incubated at 37°C in a humidified atmosphere of 10% $CO_2$ in air. Cultures at confluence were resuspended with 0.06% trypsin/1 mM diaminoethanetetra-acetic acid and passaged at 1:10 split. Viability of cells was shown by trypan blue dye exclusion to be >95%. Culture medium was replaced every 3 days and cells passaged every 6–7 days. Cell lines were regularly tested and shown to be free of mycoplasma contamination using the Hoescht fluorochrome method of Chen (Chen, 1977). Explant cultures were established by seeding healthy mixed tumour fragments at low density in tissue culture dishes in EC10 medium.

Correspondence: L.M. Franks.
Received 20 October 1983; accepted 4 January 1984.

© The Macmillan Press Ltd., 1984

*Tumour transplantation and cell inoculation*

The lower right flank was used in all experiments as the subcutaneous (s.c.) site for tumour transplants and cell inoculation. Tumours were transplanted by the implantation of pooled tumour fragments from different non-necrotic areas of tumour (to exclude any selective artefact due to structural zonal heterogeneity (Tropé, 1982; Fidler & Hart, 1981)) using a Bashford needle. Suspensions of viable single cells (cell number assessed by duplicate aliquot counts using a Coulter counter and viability by trypan blue dye exclusion) were injected in 0.1 ml of serum and antibiotic-free E4.

*Quantitation of pulmonary metastases*

Surface pulmonary metastases visualized by distending the bronchi with India ink (for details see Wexler, 1966) appeared as discrete white patches against black healthy lung tissue and were counted on separated lung lobes using a dissecting microscope. Histological sections of random lung samples confirmed the neoplastic nature of the white deposits being scored as metastases. Autopsies were done on all mice and any other apparently abnormal tissue examined histologically.

*Heterogeneity of the CMT64 cell line*

*In vitro cloning* Single cells selected from a dilute cell suspension using a 1 $\mu$l Pederson pipette, were transferred to wells of a Titertek plate (Flow Laboratories, Irvine, Scotland) and clones established from wells with single colonies.

*In vivo selection* Four different metastasising sublines of CMT64; 167, 170, 175 and 181 were sequentially selected from lung metastases in C57B/T female mice as follows: Pooled lung metastases arising from a tumour produced by CMT64/22 (culture passage no. 22) cells were transplanted s.c. and mixed fragments from the resultant s.c. tumour explanted in culture. Epithelial outgrowths were established as the first subline CMT167. Five $\times 10^6$ CMT 167/4 cells were then inoculated s.c. and after 3 weeks pooled lung metastases were again transplanted s.c. and the resultant s.c. tumour explanted as above to give rise to CMT170. Subline CMT175 was selected from CMT170, and CMT181 from CMT175 using the same method.

**Results**

*The "parent" cell line: CMT64*

The development and characterisation of the CMT64 cell line has been described (Franks *et al.*, 1976). Details of the ultrastructure of CMT64 (and of "high" metastatic sublines 167, 170, 175 and 181) and of organ distribution of metastases are to be found in the accompanying paper (Franks & Layton, 1984).

In vivo *behaviour of the cell lines*

Syngeneic C57B/T old (25 months) and young (5 months) male and female mice were used to assess the effects of age and sex of the host on the tumorigenicity of CMT64 cells. Subcutaneous inoculation of $5\times10^5$ cells gave palpable tumours in 47 of 48 mice by 8 days; after 3 weeks the mice were killed and the s.c. tumours cleanly excised and weighed. A significant increase in tumorigenicity was seen in the old mice of both sexes. The median value of s.c. tumour weights (mg) and range for the 4 groups of 12 mice were: old female: 312.5 mg (250–500); old male: 300.00 mg (100–425); young female: 100.00 mg (30–200); young male: 137.5 mg (75–250). A s.c. dose of $10^4$ cells consistently produces a local tumour, although tumours occasionally develop from fewer cells but with a greatly increased latent period. A s.c. dose of $5\times10^5$ cells produces well-defined local tumours that have metastasised to the lung by three weeks.

*Stability of the tumorigenic and metastatic phenotype*

The 'parent' CMT64 cell line and the first lung-metastases-derived subline CMT167 were examined to assess any change in tumorigenic or metastatic behaviour that may have occurred in culture. Both cell lines were compared at "10-passage" intervals ($\sim$10 weeks in culture) over 40–50 weeks. Tumorigenicity was assessed by weight of the local s.c. tumour and spontaneous metastasising behaviour by the number of surface pulmonary metastases. In this preliminary experiment only small numbers of mice were used and data are not shown as more extensive studies are in progress, but results obtained suggest that between passages 16–46 CMT64 cells displayed little difference in metastatic potential or tumorigenicity. At later passages up to 66 there appears to have been a significant increase in tumorigenic and metastatic ability. CMT167 cells, on the other hand, appeared stable in tumorigenic and metastatic behaviour over the period studied i.e. passage 6–46 although there was some indication of an increase in metastatic potential at later passages. In all experiments reported here both CMT64 and 167 were used at relatively early passages, approximately passage 24 for CMT64 and passage 15 for CMT167 to try to exclude any variation in *in vivo* behaviour due to *in vitro* induced alterations in phenotype.

### *Metastasising behaviour of clones of CMT64 and of its sublines*

Sublines of CMT64-181 derived from single cell clones were tested for their ability to metastasise. Mice were injected s.c. with $5 \times 10^5$ cells and killed 22–23 days later. The results are shown in Table I. These results show that all the cell lines had different cloning efficiencies and contained subpopulations of cells of varying metastatic potential, including one subpopulation that appears to be non-metastatic – CMT170 clone E9*.

### *Metastasis after subcutaneous inoculation of CMT64-181*

The metastasising behaviour of the parent CMT64 cell line and the sublines CMT167, 170, 175 and 181 were compared after s.c. inoculation of $5 \times 10^5$ cells into adult (4–6 months) C57 B/T female, adult athymic female Nu/Nu mice (approx. 2 months) and weanling females (3 weeks) of both strains. All mice were killed after three weeks. The results are shown in Table II. Despite variation in the numbers of metastases between individual mice, the sequentially selected sublines show much greater metastatic ability than the parent CMT64 low metaststic line irrespective of host. There appears to be an increase in metastatic *potential* with sequential selection but the size of the *expressed* increase is strongly influenced by the age and immune status of the host. The cell lines tend to be more metastatic in syngeneic mice and show greatest s.c. tumour growth in syngeneic weanlings. These results show that both cellular and host factors are involved in determining the expression of metastatic potential and are discussed later.

### *"Metastasis" after intravenous inoculation of CMT64-181*

We have used an "experimental metastasis" assay to compare the ability of CMT64-181 cells to colonise the lungs after inoculation of $5 \times 10^5$ or $5 \times 10^3$ cells in 0.1 ml of serum and antibiotic-free E4 medium into the tail veins of C57B/T female adult mice. The group receiving $5 \times 10^5$ cells quickly became ill and were killed within 10 days, by which time pulmonary metastases were too numerous to be counted (see Franks & Layton, 1984). The mice receiving $5 \times 10^3$ cells were killed 5 weeks later and the number of pulmonary metastases counted. The results are shown in Table III and suggest that the increased potential of the sublines for "spontaneous metastatic" ability is accompanied by an increased though different capacity for lung colonization.

**Table I** Metastatic behaviour of CMT64-181 and their clones in syngeneic adult mice

| *Cell line and clones* | | *Number of pulmonary metastases in individual C57 mice* | *Median* | *Range* |
|---|---|---|---|---|
| CMT64 | (×18) | 0,0,0,0,2,3,3,3,4,4,4,5,5,5,5,5,9,16. | 4.0 | (0–16) |
| CMT64/ clones: | C11/6 | 2,2,6,7,8. | 6.0 | (2–8) |
| | C12/5 | 3,11,12,14,15. | 12.0 | (3–15) |
| (cloning | E4/6 | 0,2,8,8,9. | 8.0 | (0–9) |
| efficiency | E10/4 | 0,0,2,5,14. | 2.0 | (0–14) |
| =20.83%) | A7/4 | 7,7,10,14,105. | 10.0 | (7–105) |
| CMT167 | (×24) | 2,2,5,7,12,16,21,22,22,24,27,27,28,28,30,34,34,61,63,68,77,85,90,99. | 27.5 | (2–99) |
| CMT167/10 clones: | A5/12 | 10,15,16,19,20. | 16.0 | (10–20) |
| | C2/7 | 4,5,7,8,14. | 7.0 | (4–14) |
| | C4/12 | 9,19,31,33,34. | 31.0 | (9–34) |
| | C6/10 | 19,40,46,50,61. | 46.0 | (19–61) |
| | C10/14 | 16,16,64,68. | 40.0 | (16–68) |
| (cloning | E1/14 | 12,18,25,30,37. | 25.0 | (12–37) |
| efficiency | E9/9 | 0,0,1,1,3. | 1.0 | (0–3) |
| =30.33%) | G4/13 | 3,5,6,9,21. | 6.0 | (3–21) |
| CMT170 | (×19) | 0,4,4,9,11,11,18,18,19,33,35,35,38,43,50,57,61,84,96. | 33.0 | (0–96) |
| CMT170/5 clones: | C10/8 | 4,5,18,27,53. | 18.0 | (4–53) |
| | *E9/9 | 0,0,0,0,0. | 0.0 | (0–0) |
| (cloning efficiency =12.5%) | G12/10 | 7,16,20,43,103. | 20.0 | (7–103) |
| CMT175 | (×10) | 8,10,10,16,17,17,17,21,34,49. | 17.0 | (8–49) |
| CMT175/7 clones: | A5/14 | 6,9,25,27,47. | 25.0 | (6–47) |
| | E11/12 | 6,16,16,24,54. | 16.0 | (6–54) |
| (cloning efficiency =8.33%) | | | | |
| CMT181 | (×14) | 3,4,5,6,8,10,10,17,21,21,26,28,40,46. | 13.5 | (3–46) |
| CMT181/7 clones: | A1/15 | 5,5,6,16,18. | 6.0 | (5–18) |
| (cloning efficiency =8.33%) | A5/9 | 7,8,14,18,19. | 14.0 | (7–19) |

**Table II** Effect of age and immune status of host on tumorigenicity and metastasising ability of CMT64-181 cells

| Host | Cell line | No. of mice | Median No. of pulmonary metastases | 95% confidence interval of median | Interquartile range | Median subcutaneous tumour weight (mg) | 95% confidence interval of median | Interquartile range ( ) mg | Trend statistic[a] for CMT64-CMT 181 |
|---|---|---|---|---|---|---|---|---|---|
| C57B/T | CMT64 | 18 | 4.0 | (2–5) | (2–5) | 280.0 | (239.6–384.4) | (243.0–360.7) | |
| female | CMT167 | 24 | 27.5 | (21–61) | (21–61) | 346.80 | (239.6–539.6) | (239.6–539.6) | |
| adult | CMT170 | 19 | 33.0 | (11–50) | (11–50) | 291.50 | (210.2–467.5) | (231.5–466.5) | |
| (4–6 months) | CMT175 | 10 | 17.0 | (10–34) | (10–21) | 421.25 | (276.8–479.7) | (316.2–476.0) | 1-tailed P=0.0182 |
| | CMT181 | 14 | 13.5 | (5–28) | (6–26) | 445.25 | (352.5–589.1) | (385.8–548.6) | |
| C57B/T | CMT64 | 12 | 7.0 | (1–31) | (3–25) | 592.40 | (263.7–643.6) | (311.2–630.2) | |
| female | CMT167 | 16 | 21.0 | (11–37) | (12–35) | 430.45 | (343.6–660.4) | (366.7–618.5) | |
| weanling | CMT170 | 19 | 31.0 | (18–62) | (18–62) | 537.05 | (399.5–595.9) | (399.5–595.9) | |
| (~21 days) | CMT175 | 12 | 13.5 | (8–51) | (9–50) | 531.25 | (443.5–726.8) | (464.0–646.6) | 1-tailed P=0.0003 |
| | CMT181 | 13 | 61.0 | (22–100) | (25–86) | 682.40 | (543.6–922.4) | (662.7–861.9) | |
| Nu/Nu | CMT64 | 8 | 1.5 | (0–9) | (1–2) | 144.55 | (24.2–433.6) | (130.0–230.0) | |
| female | CMT167 | 10 | 9.5 | (0–25) | (5–23) | 378.20 | (241.6–641.6) | (270.0–521.5) | |
| adult | CMT170 | 9 | 10.0 | (3–12) | (6–11) | 327.40 | (238.2–414.8) | (298.2–385.5) | |
| (7–9 weeks) | CMT175 | 9 | 10.0 | (3–100) | (5–56) | 388.90 | (167.3–928.2) | (302.2–591.4) | 1-tailed P=0.0174 |
| | CMT181 | 9 | 6.0 | (4–39) | (6–18) | 486.50 | (329.2–641.2) | (349.6–625.0) | |
| Nu/Nu | CMT64 | 10 | 0.0 | (0–7) | (0–4) | 197.35 | (87.5–428.6) | (138.1–418.0) | |
| female | CMT167 | 10 | 9.0 | (4–28) | (6–14) | 359.90 | (190.3–492.4) | (275.4–472.0) | |
| weanling | CMT170 | 13 | 23.0 | (9–48) | (9–46) | 370.50 | (170.1–463.0) | (183.8–441.0) | |
| (~23 days) | CMT175 | 13 | 18.0 | (6–46) | (8–37) | 268.80 | (175.0–507.5) | (215.3–372.0) | 1-tailed P=0.0002 |
| | CMT181 | 11 | 21.0 | (4–35) | (14–26) | 343.80 | (292.3–417.0) | (307.9–367.3) | |

[a]A test for trend in Wilcoxon rank regression model (J. Cuzick, in preparation).

**Table III** Comparison of lung colonization by CMT64–181 cells after i.v. Inoculation of $5 \times 10^3$ cells in C57 adult mice

| Cell line and passage number | Number of pulmonary deposits | Median number (Range) |
|---|---|---|
| CMT64/28 | 0, 0, 2, 2, 24 | 2.0 (0–24) |
| CMT167/19 | 2, 5, 7, 9, 17 | 7.0 (2–17) |
| CMT170/16 | 13, 20, 29, 32, 32 | 29.0 (13–32) |
| CMT175/19 | 6, 16, 91, 99, 101 | 91.0 (6–101) |
| CMT181/18 | 9, 22, 78, 166 | 50.0 (9–166) |

### Discussion

There is evidence that metastasis is a selective and not a random process implying the pre-existence of cell subpopulations of varying metastatic potential within a primary tumour (Poste & Fidler, 1980; Talmadge, 1983). We have confirmed this metastatic cellular heterogeneity since "high" metastatic sublines have been selected from the "low" metastatic CMT64 parent line. Other workers have obtained similar results (e.g. Fidler & Cifone, 1979; Neri *et al.*, 1982), although Weiss (Weiss *et al.*, 1983) points out that the subpopulation hypothesis may not be a general rule.

Poste *et al.* (1982) using B16 melanoma cell lines, have shown that cellular heterogeneity is not restricted to primary tumours but exists to a more limited degree in "spontaneous" and "experimental" metastases.

The uncloned parent line and the sublines selected from pooled lung metastases used in our metastasis assays are heterogeneous (see Table I) and have remained relatively stable since their initiation. The variation in expressed metastatic potential between individual mice may thus be due to variations in host response against different cell subpopulations. In our experiments the use of heterogeneous sublines, possibly stabilised by clonal interactions (Poste *et al.*, 1981), may have been advantageous, since although phenotypic drift may occur this instability is often much greater in clonally derived sublines (Hart & Fidler, 1981). The existence of metastatic heterogeneity and evidence for stabilisation of heterogeneous cell populations by clonal interactions support the use of well characterised cellular subpopulations with defined stable metastatic capabilities isolated from the original heterogeneous tumour cell population (Hart & Fidler, 1981). It is also essential that experimental metastasis models reflect as closely as possible clinical metastatic disease. Unfortunately many experimental systems do not meet (in contrast to CMT64 – see conclusion) the basic requirements of a suitable model. (For a detailed discussion of these problems, see Hewitt 1976, 1980; Poste, 1982.)

Another problem associated with choice of models for metastasis is that many of the tumours used do not metastasise spontaneously. These systems depend on the i.v. inoculation of very large numbers of single tumour cells directly into the blood stream at one time, 'experimental metastasis'; certainly not representative of the normal metastatic process in clinical cancer. In the CMT64 system the cells spread in solid cords extending from the main tumour and dissemination of single cells is not common. The frequency with which small numbers of tumour cells are found in the blood in man without development of metastases (Malmgren, 1967) and a similar lack of metastases in patients in whom ascitic fluid containing small clumps of tumour cells had been diverted into the blood-lymphatic system (Tarin, personal communication) together with data showing that the majority of tumour cells reaching the circulation do not survive (Weiss *et al.*, 1982) would seem to make it unlikely that single tumour cells or even small clumps will produce distant metastases. Even with highly malignant experimental tumours, a substantial number of tumour cells must be inoculated before a tumour will develop, e.g. $10^4$ for CMT64 cells. Our results have shown that the different metastatic abilities shown by the CMT64-181 lines after s.c. inoculation are only partially paralleled by lung colonising ability after i.v. inoculation and Stackpole (1981) has suggested that organ colonisation after injection of cells directly into blood vessels does not necessarily predict spontaneous metastatic potential from s.c. transplants, and has reported the existence of two distinct cell populations in the B16 melanoma: lung colonizing and lung metastasising.

In our system both cellular and host factors are involved in determining local tumour growth and metastasis since the cell lines differ in tumorigenicity and metastatic capacity depending on the age and immune status of the host. All cell lines produced larger tumours in syngeneic weanlings, and in general were more metastatic in syngeneic hosts. This was unexpected since we felt that the immunoincompetence of Nu/Nu mice might allow greater expression of metastatic potential, although other workers have suggested that the nude mouse may be less susceptible to the development of metastases from murine tumours (e.g. Skov *et al.*, 1976; Prehn & Lappé, 1971; Fidler *et al.*, 1977). Our results have shown that Nu/Nu weanling mice appear to allow greater expression of the metastatic potential of CMT170, 175 and 181 cell lines than the Nu/Nu adults, and that all cell lines metastasise to a greater extent in syngeneic adults than Nu/Nu adult hosts. Hanna (1982) has reviewed evidence suggesting that an active T cell independent defence mechanism might be responsible for the low incidence of tumour metastasis in adult nude mice and that low natural killer (NK) cell activity in weanling three week old mice may support the expression of metastatic potential.

We have not been able to find any direct correlation between behaviour in *in vitro* assays and metastatic behaviour of the cell lines or their clones and a preliminary investigation of cell surface proteins (Steele *et al.*, 1983) has revealed only small differences between the cell lines although a more extensive study is in progress. It is thus possible that the significant differences in metastatic behaviour may be due, in part, to changes induced by the host environment.

In conclusion, we feel that the lung tumour model described here is particularly useful for the experimental investigation of metastasis, since the original tumour was spontaneous, is transplantable to syngeneic immunocompetent hosts and the cell lines of varying metastatic ability are epithelial, stable, well differentiated, probably weakly immunogeneic, metastasise within 3 weeks in both syngeneic C57B/T and Nu/Nu mice and colonize the lungs in experimental metastasis assays. The CMT64 system will be of great value in the study of the complex cellular and host factors interacting in the process of metastasis and may be of use in the screening of therapeutic agents.

### References

CHEN, T.R. (1977). In situ detection of mycoplasma contamination in cell cultures by fluorescent Hoescht 33258 stain. *Exp. Cell Res.*, **104**, 255.

FIDLER, I.J. & CIFONE, M.A. (1979). Properties of metastatic and non-metastatic cloned subpopulations of an ultraviolet light induced murine fibrosarcoma of recent origin. *Am. J. Pathol.*, **97**, 633.

FIDLER, I.J., GERSTEN, D.M. & RIGGS, C. (1977). Relationship of host immune status to tumor cell arrest, distribution and survival in experimental metastasis. *Cancer*, **40**, 46.

FIDLER, I.J. & HART, I.R. (1981). Biological and experimental consequences of the zonal composition of solid tumours. *Cancer Res.*, **41**, 3266.

FRANKS, L.M., CARBONELL, A.W., HEMMINGS, V.J. & RIDDLE, P.N. (1976). Metastasizing tumours from serum-supplemented and serum-free cell lines from a C57BL mouse lung tumour. *Cancer Res.*, **36**, 1049.

FRANKS, L.M. & LAYTON, M.G. (1984). Ultrastructural tumour differentiation and organ specificity in high and low metastatic lines from a mouse lung carcinoma. *Br. J. Cancer*, **49**, 423.

HANNA, N. (1982). Role of natural killer cells in control of cancer metastasis. *Cancer Met. Rev.*, **1**, 45.

HART, I.R. & FIDLER, I.J. (1981). The implications of tumour heterogeneity for studies on the biology and therapy of cancer metastasis. *Biochem. Biophys. Acta*, **651**, 37.

HEWITT, H.B. (1976). Projecting from animal experiments to clinical cancer. In *Fundamental Aspects of Metastasis*, p. 344. (Ed. Weiss) North Holland Publishing Company.

HEWITT, H.B. (1980). Animal tumour models: The intrusion of artefacts. In *Metastasis: Clinical and Experimental Aspects*, p. 18. (Eds. Hellmann *et al.)* The Hague: Martinus Nijhoff.

MALMGREN, R.A. (1967). Studies of circulating cancer cells in cancer patients. In *UICC Monograph Series 6, Mechanisms of Invasion of Cancer*, p. 108. (Ed. Deroix) New York: Springer-Verlag.

NERI, A., WELCH, D. KAWAGUCHI, T. & NICOLSON, G.L. (1982). Development and biologic properties of malignant cell sublines and clones of a spontaneously metastasizing rat mammary adenocarcinoma. *J. Nat Cancer Inst.*, **68**, 507.

POSTE, G. (1982). Experimental systems for analysis of the malignant phenotype. *Cancer Met. Rev.*, **1**, 141.

POSTE, G. & FIDLER, I.J. (1980). The pathogenesis of cancer metastasis. *Nature*, **283**, 139.

POSTE, G., DOLL, J., BROWN, A.E., TZENG, J. & ZEIDMAN, I. (1982). Comparison of the metastatic properties of B16 melanoma clones isolated from cultured cell lines, subcutaneous tumours and individual lung metastases. *Cancer Res.*, **42**, 2770.

POSTE, G., DOLL, J. & FIDLER, I.J. (1981). Interactions among clonal subpopulations affect stability of the metastatic phenotype in polyclonal populations of B16 melanoma cells. *Proc. Nat. Acad. Sci.*, **78**, 6226.

PREHN, R.T. & LAPPÉ, M.A. (1971). An immune stimulation theory of tumour development. *Transplant Rev.*, **7**, 26.

ROWLATT, C., FRANKS, L.M., SHERIFF, M.U. & CHESTERMAN, F.C. (1969). Naturally occurring tumours and other lesions of the digestive tract in untreated C57BL mice. *J. Nat Cancer Inst.*, **43**, 1353.

SKOV, C.B., HOLLAND, J.M. & PERKINS, E.H. (1976). Development of fewer tumour colonies in lungs of athymic mice after intravenous injection of tumour cells. *J. Nat Cancer Inst.*, **56**, 193.

STACKPOLE, C.W. (1981). Distinct lung-colonizing and lung-metastasizing cell populations in B16 mouse melanoma. *Nature*, **289**, 798.

STEELE, J.G., ROWLATT, C., SANDALL, J.K. & FRANKS, L.M. (1984). Cell surface properties of high and low metastatic cell lines selected from a spontaneous mouse lung carcihoma. *Int. J. Cancer*, **32**, 769.

TALMADGE, J.E. (1983). The selective nature of metastasis. *Cancer Met. Rev.*, **2**, 25.

TROPÉ, C. (1982). Different susceptibilities of tumour cell subpopulations to cytotoxic agents. In *Design of Models for Testing Cancer Therapeutic Agents*, p. 64 (Eds. Fidler & White). Van Nostrand Reinhold: Litton Bionetics Workshop.

WEISS, L., MAYHEW, E., GLAVES RAPP, D. & HOLMES, J.C. (1982). Metastatic inefficiency in mice bearing B16 melanomas. *Br. J. Cancer*, **45**, 44.

WEISS, L., HOLMES, J.C. & WARD, P.M. (1983). Do metastases arise from existing subpopulations of cancer cells? *Br. J.Cancer*, **47**, 81.

WEXLER, H. (1966). Accurate identification of experimental pulmonary metastases. *J. Natl Cancer Inst.*, **36**, 641.